# Sanctioned countries in the global COVID-19 vaccination campaign: the forgotten 70%

**DOI:** 10.1186/s13031-021-00404-2

**Published:** 2021-09-17

**Authors:** Karl Blanchet, Grégoire Mallard, Erica Moret, Jin Sun

**Affiliations:** 1 Geneva Centre of Humanitarian Studies, Geneva, Switzerland; 2 Faculty of Medicine, University of Geneva, Geneva, Switzerland; 3 Graduate Institute of International and Development Studies, Geneva, Switzerland

## Abstract

COVAX, the global initiative on COVID-19 vaccines, has set the target of achieving a COVID-19 vaccination coverage of up to 30% of the population of 135 countries in 2 years. Here, we argue that COVAX should anticipate important and unforeseen challenges with regard to sanctioned countries. For those, COVAX needs to provide a higher percentage of the vaccines. Otherwise, the problems of vaccination will linger, risking the advent of new variants and preventing an effective global response in reigning the pandemic under control.

## Background

COVAX, the global initiative on COVID-19 vaccines, has set the target of achieving a COVID-19 vaccination coverage of up to 30% [1] of the population of 135 countries in 2 years [2], with advance commitment to ensure access for 92 low- and middle-income countries (LMICs) [3]. This appears to be an ambitious and welcome objective.

Today, COVAX faces criticism mostly for the delays experienced by the implementation of its vaccination program. Indeed, as UNICEF shows [2], COVAX has shipped up to 68.8 million doses in 81 of the 92 LMICs, indicating delivery of only 3.8% of the promised 1.8 billion doses. Till now, eleven LMICs have not received any doses from COVAX. The main reasons for such a failure are however external to COVAX, and related to supply side issues like over-procurement by high income countries [4], or vaccine and vaccine material export restrictions imposed by high-income countries (HICs) like the US [5], leaving few stocks for LMICs. As the vaccination rates in HICs sits at about 40% [6], there is reason to be hopeful that COVAX will be able to support LMICs in time.

Here, we argue that COVAX should anticipate additional challenges with regard to sanctioned countries. Currently, the UN, US and EU and others impose sanctions on over 20 countries around the world. While some of these sanctions are highly targeted in nature (such as arms embargoes, travel bans and individual asset freezes), and thus unlikely to influence certain countries’ access to the vaccine, others represent *de-facto* comprehensive sanctions regimes, whose return results from the multiplying effect of overlapping sanctions regimes (sometimes alongside counterterrorism and anti-money-laundering regulations), the adoption of broad sectoral sanctions (including those on finance and energy sectors in a dozen of countries) and the growing reluctance seen in the international banking sector to service transactions to so-called “high-risk” countries (known as over-compliance or ‘de-risking’) even for vaccine procurement.

While COVAX assumes that all LMICs will be able to procure 70% of the remaining doses, it has thus ignored the three specific difficulties that sanctioned countries run into when they seek to procure such type of goods. First, COVAX may experience problems when delivering the vaccine to sanctioned countries, such as Iran, North Korea, Syria, Venezuela, Afghanistan and Myanmar, due to hindered access to goods and services including temperature-controlled equipment, fuel, software, insurance or logistics operators. Relatedly, COVAX may not be able to deliver or administer vaccines to certain groups within de-risked countries, such as members of designated terrorist organisations (like Hezbollah) and other non-state armed groups.

Second, most sanctioned countries are not in a position to procure the remaining 70% of the COVID vaccines. Exporters or their banks may not authorize the transactions with them, again due to de-risking. After a wave of heavy sanctions enforcement actions by the US Treasury, seeing global banks paying fines entering into the billions of US dollars, all global banks increasingly avoid any transactions with US-sanctioned countries, like Iran and Venezuela [7]. UNICEF data [8] shows that while non-sanctioned countries directly purchase from multiple suppliers, global pharmaceutical companies declined the sales to sanctioned countries: none of them signed a supply agreement with Syria, Afghanistan, North Korea, Venezuela or Iran, and only one supplied to Myanmar but only for 25% of the population.

Third, bilateral donations, sometimes described as “vaccine diplomacy”, have been delivered to some sanctioned countries by donors. It may make the impression in the international community that COVAX should concentrate on servicing the non-sanctioned developing countries and leave the delivery of vaccines to sanctioned countries to big powers. UNICEF data (Table 1) indicates that major economies, such as China, India, the US, Japan, Russia, and France are active in bilateral donations to specific countries, but the recipients are not those in most urgent humanitarian need.

**Table 1:** Covid-19 vaccine donation chart [9]

Donors	# (million)	%	Recipients	# (million)	Source
China (C)	23.97	38.9	Pakistan	4	C, U
India (I)	8.88	14.4	China Taipei	3.74	J, U
US (U)	8.51	13.8	Bangladesh	3.2	C, I, U
Japan (J)	8.37	13.6	Nepal	2.9	C, I
Switzerland	4	6.5	Myanmar	2.2	C, I
MTN Group	1.76	2.9	Cambodia	2.2	C
Australia	1.11	1.8	Laos	1.6	C
Russia	1.04	1.7	Afghanistan	1.6	C, I
France	0.82	1.3	Sri Lanka	1.6	C, I
Total	61.6	100.0		39.6	

## Conclusion

Because of these three reasons, we estimate that in 2 years, with COVAX setting a target of procuring 30% of the needed vaccines in LMICs, about 60 to 70% of the adult population in the non-sanctioned developing countries could be vaccinated, but that less than 20% of the total adult population will be vaccinated in the sanctioned developing world. We thus urge COVAX to rethink its targets and set two different kinds of programmes depending on whether LMICs face risks of financial exclusion because of sanctions. For those, COVAX needs to provide a higher percentage of the vaccines. Otherwise, the problems of vaccination will linger, risking the advent of new variants and preventing an effective global response in reigning the pandemic under control.

## Data Availability

Data are available upon reasonable request. Data underlying this publication are available upon request to correspondence, UNICEF Covid-19 vaccine market dashboard: https://www.unicef.org/supply/covid-19-vaccine-market-dashboard, and, Bloomberg COVID vaccine tracker: https://www.bloomberg.com/graphics/covid-vaccine-tracker-global-distribution/.

## Abbreviations

COVAX: COVID-19 vaccines global access initiative
HICs: High-income countries
LMICs: Low- and middle-income countries
UNICEF: UN Children Foundation
WHO: World Health Organization
